# Pathological microbial niches in diabetic foot ulcers: a host-microbe framework for impaired repair and niche-based stratification

**DOI:** 10.3389/fcimb.2026.1926191

**Published:** 2026-10-02

**Authors:** Min Leng, Zairong Wei

**Affiliations:** 1 Department of Burns and Plastic Surgery, Affiliated Hospital of Zunyi Medical University, Zunyi, Guizhou, China; 2 Dazhou Central Hospital, Dazhou, Sichuan, China; 3 The Collaborative Innovation Center of Tissue Damage Repair and Regeneration Medicine, Zunyi Medical University, Zunyi, Guizhou, China; 4 Guizhou Biofabrication Laboratory, Affiliated Hospital of Zunyi Medical University, Zunyi, Guizhou, China

**Keywords:** biofilm, diabetic foot ulcer, host-microbe interaction, multi-omics, non-healing inflammation, pathological niche, spatial omics, wound microbiome

## Abstract

Diabetic foot ulcers (DFUs) are commonly described through the clinical triad of hyperglycaemia, ischaemia and infection. That language is useful for bedside decision-making, but it does not fully explain why some wounds remain inflamed and open despite debridement, offloading, antimicrobial therapy, vascular assessment and modern wound care. Here we develop a conceptual review framework in which many non-healing DFUs are interpreted as pathological microbial niches: local tissue regions where microbial persistence and host repair failure reinforce each other. Hyperglycaemia, impaired perfusion, hypoxia, neuropathic pressure injury, necrotic material and extracellular matrix degradation generate ecological space for microbial survival. Microbial products, biofilm-like spatial organisation, strain-level traits, fungi and polymicrobial interactions then amplify neutrophil extracellular traps, inflammasome signalling, protease release, fibroblast dysfunction, endothelial impairment and defective re-epithelialisation. The result is not a linear sequence from colonisation to infection to inflammation, but a self-reinforcing loop in which microbial adaptation and host non-healing programs become difficult to separate. We summarise evidence from chronic wound microbiome studies, biofilm histology, strain-level and fungal analyses, host transcriptomics, single-cell data and emerging spatial omics, while emphasising the boundaries of each evidence type. The central question is not whether microbes can be detected in DFU tissue, but whether they are viable, metabolically active, spatially organised and positioned close enough to host cells to alter repair behaviour. We propose an operational definition of the pathological microbial niche and a translational scheme for biofilm-dominant, ischaemia-dominant, protease-dominant, senescence-dominant and immune-resolution-failure wound patterns. This framing may help move DFU microbiome research from descriptive association toward mechanism-based stratification and more rational combinations of source control, vascular optimisation, antimicrobial therapy and repair-directed treatment.

## Highlights

- A DFU microbial signal is most informative when it can be linked to microbial activity, tissue location and neighbouring host repair state.
- The pathological niche concept is narrower than the general wound microbiome and broader than clinical infection; it focuses on biological coupling between microbes and impaired repair.
- High-throughput sequencing should be paired with microbial load, histology, spatial localisation, contamination control and longitudinal clinical metadata.
- Niche-based stratification may support rational combinations of debridement, offloading, vascular optimisation, antimicrobial treatment and repair-directed therapy.

## 1 Introduction

Diabetic foot ulceration remains one of the most difficult complications of diabetes to manage. It brings prolonged treatment, recurrent infection, hospitalisation, limb loss, high cost of care and excess mortality (Armstrong et al., 2023; Brem and Tomic-Canic, 2007; Frykberg and Banks, 2015). Even when a wound closes, the problem is often not over; many DFUs behave more like a chronic disease in remission than a resolved injury, with substantial recurrence after healing (Armstrong et al., 2017). Current guidelines rightly emphasise risk assessment, offloading, infection diagnosis, debridement, vascular evaluation, wound-bed preparation and selected adjunctive therapies (Bus et al., 2024; Chen et al., 2024; Schaper et al., 2024; Senneville et al., 2024). Yet a familiar clinical puzzle remains: some wounds receive appropriate care and still fail to progress. This gap suggests that conventional labels such as infection, ischaemia or poor glycaemic control capture important risks but do not fully describe the local biology of non-healing.

The difficulty is partly conceptual. Clinical risks are often listed side by side, as if each acted independently (Guo and Dipietro, 2010). In the wound bed they are entangled. Hyperglycaemia alters immune metabolism, endothelial function and nutrient conditions for microorganisms. Poor perfusion restricts oxygen delivery, immune cell trafficking and antibiotic access. Neuropathy and repeated pressure injury generate callus, tissue disruption and necrotic material. Antibiotic exposure, cleaning, debridement and dressing changes add further selection pressure. The wound therefore becomes more than damaged tissue. It becomes a local ecological setting in which microorganisms can persist while host cells remain caught between inflammation and incomplete repair.

Classic wound-healing biology describes an ordered transition from haemostasis and inflammation to proliferation, matrix deposition, epithelial closure and remodelling (Gurtner et al., 2008; Eming et al., 2014; Rodrigues et al., 2019; Singer and Clark, 1999; Falanga, 2005; Wilkinson and Hardman, 2020). DFUs disrupt this timing at several levels. Keratinocytes at the wound edge may remain activated but poorly migratory. Fibroblasts may produce inflammatory mediators and proteases rather than reconstruct a stable extracellular matrix. Neutrophils and macrophages may remain abundant but fail to clear microorganisms, remove debris and terminate danger signalling. The same microbial product that would trigger a short protective response in an acute wound can sustain tissue damage when the local environment is hypoxic, senescent and matrix-degraded.

We use the term pathological microbial niche for this kind of local wound environment. The term is meant to be precise. It is narrower than the general wound microbiome because it does not refer to all microbes detected by culture or sequencing. It is broader than clinical infection because it includes microbial activity that may shape repair even when overt infection is not the only dominant clinical problem. It asks a practical set of questions: where are the microbes, are they active, what tissue compartment do they occupy, and do neighbouring host cells show programs consistent with repair failure? Earlier chronic-wound microbiome work already showed that wound communities are diverse, spatially structured and clinically variable rather than simple extensions of culture-positive infection (Bjarnsholt et al., 2008; Dowd et al., 2008; Price et al., 2009; Gardner et al., 2013). The niche concept builds on that observation and gives it a mechanistic focus.

To make this contribution explicit, the present framework differs from earlier biofilm-centred chronic-wound hypotheses in four operational ways. First, it defines a pathological microbial niche by simultaneous evidence of location, microbial activity, host repair state, clinical context and validation rather than by microbial persistence alone. Second, it ranks evidence from association to activity, spatial coupling and temporal or perturbational support. Third, it states discriminating predictions and potential falsifiers, including the alternative that microbial and host changes may be parallel consequences of perfusion failure rather than coupled drivers. Fourth, it links the framework to research stratification and treatment sequencing. The intention is therefore not to rename a familiar chronic-wound biofilm idea, but to specify how a niche model could be tested and used.

This article is framed as a conceptual review because the current literature is not yet strong enough to support a single linear causal model. We therefore do not argue that all non-healing DFUs have the same microbial driver, nor that microbial eradication is always the appropriate aim. Instead, we propose that the most informative studies will test whether microbial persistence, host danger signalling and defective repair programs co-localise in time and space. This shift may help the field move from descriptive microbiome association toward clinically useful stratification. The pathological niche framework and the proposed spatial multi-omics workflow are illustrated in **Figures 1** and **2**, respectively.

**Figure 1:**
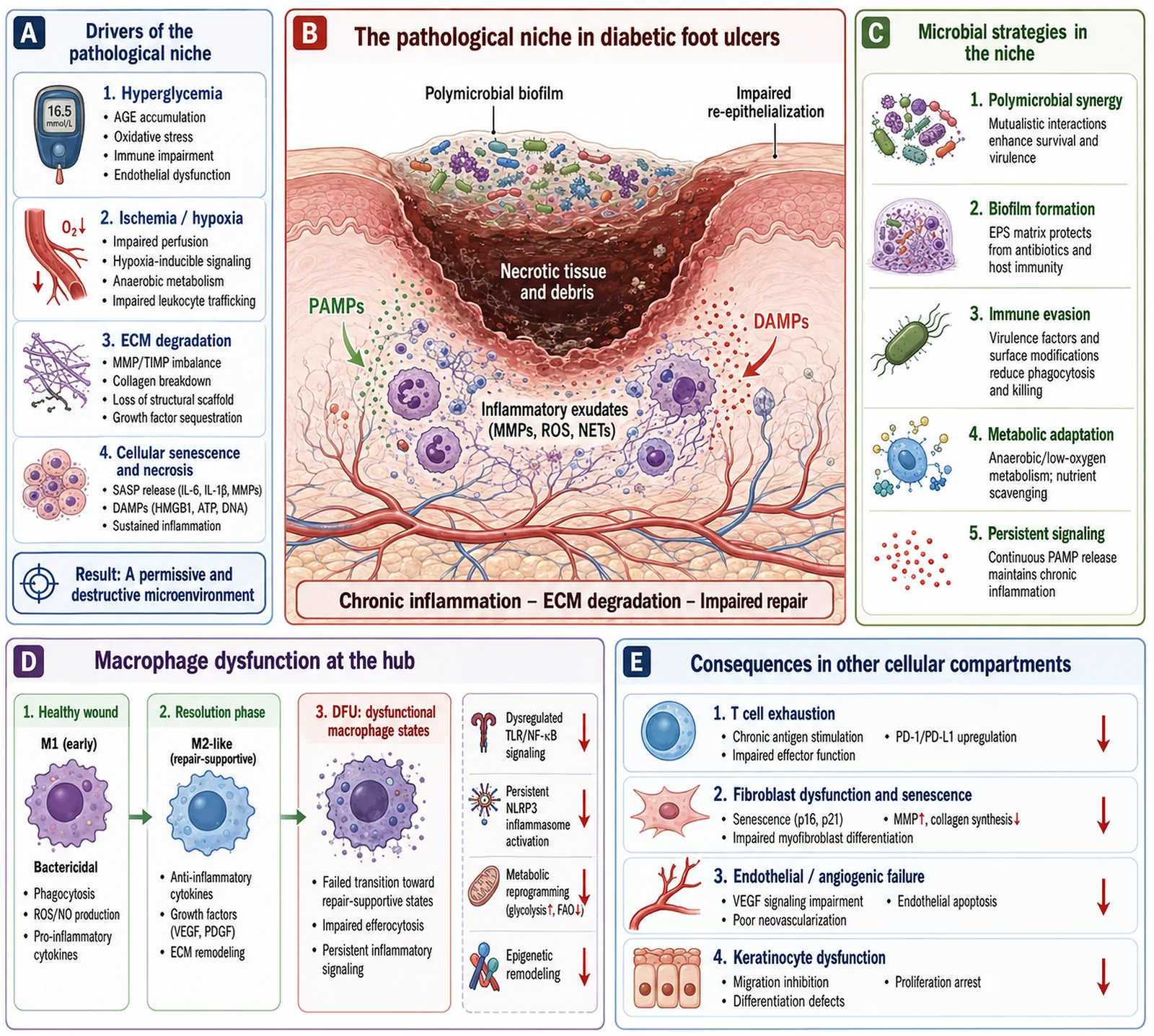
Pathological niche-driven microbe-host interactions in diabetic foot ulcers. **(A)** Drivers of the pathological niche; **(B)** the pathological niche in diabetic foot ulcers; **(C)** microbial strategies in the niche; **(D)** macrophage dysfunction at the hub; and **(E)** consequences in other cellular compartments. Metabolic, vascular and structural abnormalities in diabetes create a permissive wound microenvironment for microbial persistence. Within this niche, polymicrobial biofilms interact with necrotic tissue, damaged extracellular matrix and immune cells, generating sustained PAMP and DAMP signals. These signals amplify chronic inflammation, extracellular matrix degradation, impaired macrophage resolution, fibroblast dysfunction, angiogenic failure and defective re-epithelialization. The model emphasizes that microbial presence alone is insufficient to explain non-healing; rather, spatially organized microbial activity and host repair failure jointly maintain the pathological niche.

**Figure 2:**
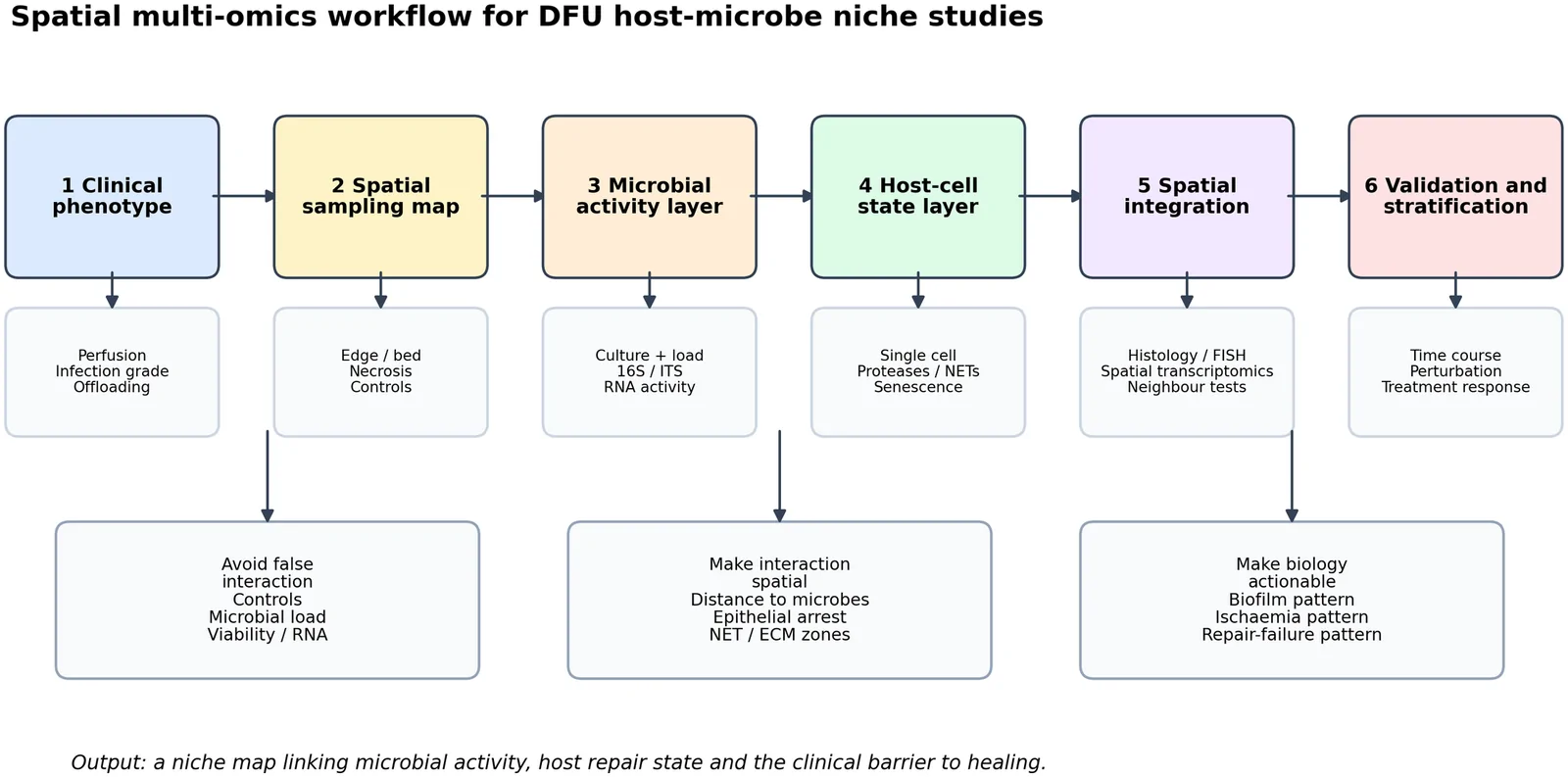
Spatial multi-omics workflow for reconstructing host-microbe causal chains in diabetic foot ulcers. Spatial transcriptomics, microbial localization, host cell-state mapping, histopathology and neighbourhood analysis can be integrated to identify microbial hotspots, host repair programs and distance-dependent interactions. This workflow separates microbial presence from spatially organized activity and links niche drivers to microbial organization and host repair failure.

## 2 Defining a pathological microbial niche in DFU wounds

A pathological microbial niche is not synonymous with colonisation, infection or a positive sequencing read. It is a spatially restricted wound microenvironment in which microbial persistence and host non-healing programs are mutually reinforcing. The definition has four components. First, microorganisms must be present in a relevant tissue compartment rather than merely detected on the surface or introduced as contamination. Second, there should be evidence of viability, metabolic activity, biofilm-like organisation, strain-level virulence or microbial products capable of stimulating host pathways. Third, neighbouring host cells should show programs compatible with repair failure, such as NET excess, inflammasome activation, protease-dominant matrix degradation, inflammatory fibroblast states, epithelial arrest, endothelial dysfunction or senescence. Fourth, the microbial and host signals should be interpreted together with clinical barriers including perfusion, pressure, depth, necrosis, antibiotics and duration.

This operational framing is useful because it separates three questions that are often conflated. The first is diagnostic: is the wound clinically infected and does it require systemic antimicrobial therapy or surgical source control? The second is ecological: what microbial communities, biofilm structures or fungal components persist in the wound? The third is mechanistic: are those microbial signals spatially and temporally coupled to host repair failure? A wound can be clinically infected without a carefully mapped niche study, and a non-severely infected wound may still contain microbial products and damaged tissue signals that maintain local inflammation. The niche concept therefore complements clinical classification rather than replacing it.

The concept also prevents the opposite error: assuming that every detected microbe is harmful. A chronic wound can contain organisms that are passengers, contaminants, opportunists, pathobionts or even potential repair-modifying commensals. For example, Alcaligenes faecalis, often recovered from chronic wounds but rarely treated as a primary pathogen, was reported to correct aberrant MMP expression and promote re-epithelialisation in diabetic wounds (White et al., 2024). Such findings complicate an eradication-only narrative. The biological issue is microbial function in a particular host and spatial context.

## 3 Formation of the pathological niche: diabetic tissue pressures

A pathological microbial niche does not appear suddenly. It emerges as the tissue microenvironment changes. Normal repair requires a timed transition from inflammatory clearance to proliferation and remodelling. In DFUs, systemic diabetic biology meets local tissue damage and distorts this sequence. Wound fluid accumulates inflammatory mediators, degraded matrix fragments, exudate proteins and necrotic debris. Oxygen delivery, immune clearance and repair-cell migration are compromised. These are precisely the conditions under which microbial persistence and biofilm tolerance become easier to sustain.

Hyperglycaemia is often treated as background information in DFU studies, but biologically it is an active selection pressure. In host cells, it promotes advanced glycation end-product signalling, oxidative stress, endothelial dysfunction and impaired immune activity (Huang et al., 2023; Rodrigues et al., 2019). Keratinocytes migrate less efficiently, fibroblasts deposit matrix less effectively, endothelial cells show weaker angiogenic responses and phagocytes kill less well. Microorganisms encounter the same environment from the opposite side: altered nutrients, reduced host clearance and changes in virulence pressure. In diabetic skin infection models, loss of nutritional immunity can promote *Staphylococcus aureus* virulence (Thurlow et al., 2020). High glucose therefore weakens the host and, at least for some organisms, improves the odds of persistence.

Ischaemia and hypoxia deepen the niche. Reduced perfusion limits oxygen-dependent killing, antibiotic delivery and recruitment of repair cells. Hypoxic and exudative zones can favour facultative anaerobes, anaerobes and mixed communities that tolerate steep oxygen and nutrient gradients. The resulting inflammation is not absent; it is often conspicuous. Chemokines, proteases and neutrophils may be present, but microbial clearance, removal of necrotic tissue and mature granulation tissue formation do not necessarily follow. Many DFUs therefore look less like immune silence than low-efficiency inflammation.

Importantly, this relationship should not be read as one-way. Hypoxic tissue can select for anaerobic or low-oxygen-tolerant communities, but dense microbial growth and biofilm-like aggregation can also consume oxygen and steepen local gradients. The model therefore treats hypoxia as both an upstream driver and a possible downstream consequence of microbial metabolic activity. A perfusion-centred alternative remains plausible in some wounds: poor oxygen delivery may independently produce epithelial arrest and microbial persistence without direct microbial-host coupling. Distinguishing these possibilities requires spatial and temporal evidence rather than taxonomy alone.

Neuropathy and pressure injury provide repeated local trauma. Callus formation, shear stress and unnoticed microtrauma generate tissue disruption and necrotic material. Even a well-selected antimicrobial regimen may not resolve this ecological driver if mechanical injury continues to create new tissue damage. Offloading should therefore be viewed not only as a biomechanical intervention but also as a way of reducing recurrent DAMP production and ecological space for microbial survival.

Extracellular matrix failure gives the niche a physical scaffold. The matrix is not only a route for keratinocyte and fibroblast movement; it can also become an attachment surface for microorganisms. Excess matrix metalloproteinase activity, weak tissue inhibitor control and poor collagen deposition push the wound bed toward degradation rather than reconstruction, a central feature of abnormal remodelling (Xue and Jackson, 2015). Matrix fragments, fibrin, exudate proteins and necrotic debris expose adhesion sites and contribute damage-associated molecular patterns. Cellular senescence can add another layer. DFU tissue shows increased expression of senescence-associated molecules, including CDKN1A, CXCL8, IGFBP2, IL1A, MMP10 and SERPINE1 (Yu et al., 2024). Together, these changes make the tissue itself a scaffold for microbial persistence and inflammatory self-maintenance. These host- and microbial-side components are summarized in **Table 1**.

**Table 1:** Components that make a pathological microbial niche in DFU wounds.

Niche component	Host-side consequence	Microbial-side consequence	Why it matters for non-healing
Hyperglycaemia and oxidative stress	Keratinocyte migration, endothelial repair and phagocyte killing are weakened.	Nutrient availability and stress selection may favour tolerant phenotypes.	The host loses clearance capacity while selected microbes gain ecological advantage.
Ischaemia and hypoxia	Oxygen-dependent killing, immune recruitment and repair-cell metabolism decline.	Low-oxygen gradients and biofilm tolerance become more stable.	Inflammation can remain visible but ineffective.
Neuropathy, callus and pressure injury	Repeated microtrauma generates tissue disruption and DAMPs.	New damaged surfaces and exudate maintain ecological space.	Mechanical injury can rebuild the niche after apparently adequate treatment.
Necrosis, exudate and damaged ECM	DAMPs, proteases and inflammatory wound fluid accumulate.	Adhesion surfaces and nutrient sources increase.	Tissue debris becomes both microbial shelter and inflammatory fuel.
ECM breakdown and cellular senescence	Matrix scaffolds fail; SASP sustains chemokines and proteases.	Fibrin, collagen fragments and matrix proteins support adherence.	Repair failure and microbial persistence reinforce each other.
Antibiotic exposure and repeated debridement	Inflammation may be partially reduced but tissue architecture remains unstable.	Community composition may shift rather than disappear, including bacterial-fungal imbalance.	Treatment can change the niche without necessarily resolving it.

## 4 Microbial persistence: beyond the presence of bacteria

Early DFU microbiology often treated microbial relevance as a matter of culture positivity or dominance by a named pathogen. Culture is still essential in infected wounds, especially for antimicrobial selection. It is less able, however, to describe the ecology of chronic wound communities. Sequencing studies show that chronic wounds may contain complex bacterial communities that change over time, and that community stability can be associated with poor healing (Loesche et al., 2017; Misic et al., 2014). Longitudinal DFU profiling also links microbial dynamics with healing, deterioration and amputation trajectories (Sachdeva et al., 2026). This fits with the broader view that the wound microbiome is not fixed but shaped by time, treatment, moisture, perfusion and tissue damage (Liu et al., 2020).

Species names alone can be misleading. Strain-level variation affects virulence, resistance, host response and therapeutic efficacy. In diabetic wounds, strain- and species-level variation has been associated with clinical outcomes and response to therapy (Kalan et al., 2019). The implication is straightforward but important: two wounds labelled with the same species may not contain the same biological problem. Experimental work also suggests that bacterial fitness in chronic wounds may depend more on stress tolerance and high-density growth than on classical virulence or biofilm functions alone (Morgan et al., 2019). When the question is whether microbes contribute to non-healing, species-level taxonomy is a starting point, not an answer.

Biofilm biology helps explain why persistence can be spatial rather than simply quantitative. Biofilms are frequently detected in chronic wounds, and histological studies show bacteria embedded in extracellular polymeric material rather than freely distributed (James et al., 2008; Malone et al., 2017). Such structures create gradients of oxygen, metabolites and antimicrobial penetration. Organisms can also be non-randomly distributed within chronic wound tissue, with different taxa occupying distinct microenvironments (Fazli et al., 2009). This organisation may support tolerance, immune evasion and interspecies cooperation. It does not mean that every poorly healing DFU contains a clinically visible mature biofilm. It does mean that tissue organisation must be considered when antimicrobial therapy appears appropriate but response remains poor.

To avoid terminology drift, we use biofilm in a strict sense only when tissue-based or imaging evidence supports organised microbial aggregates embedded in extracellular polymeric material or an equivalent matrix-rich structure. When such structural evidence is lacking, we use biofilm-like organisation, microbial aggregates or persistence pattern to indicate a plausible but unproven spatial arrangement. Sequencing enrichment, taxonomic dominance or poor antimicrobial response alone is therefore insufficient to label a finding as mature biofilm. This distinction matters because a biofilm claim implies a higher evidence threshold than microbial persistence.

Multispecies communities may stabilise the niche further. S. aureus, Streptococcus spp., *Pseudomonas aeruginosa*, anaerobes and other organisms may occupy different oxygen and nutrient zones. They can interact through metabolic cross-feeding, competition, quorum sensing, resistance exchange or modulation of host inflammation. *Pseudomonas aeruginosa*, for instance, can inhibit diabetic wound healing through type III secretion system-dependent mechanisms (Goldufsky et al., 2015). Multi-omics analysis of diabetic foot infections also suggests that severity reflects microbial activity together with host inflammatory or stress programs, rather than total microbial burden alone (Radzieta et al., 2021). The wound community is therefore better read as an ecological system than as a simple list of organisms.

Obligate anaerobes require explicit attention in DFU niche studies. Routine culture may underestimate them because oxygen exposure during sampling and transport, prior antimicrobial treatment and conventional aerobic workflows can reduce recovery. Low-oxygen wound compartments may nevertheless allow obligate anaerobes and facultative anaerobes to occupy deeper necrotic or exudative zones, where they may contribute to proteolysis, malodour, tissue breakdown or polymicrobial cross-feeding. The point is not that anaerobes are always dominant, but that their under-detection can bias the interpretation of a hypoxia-centred niche. Metagenomics, metatranscriptomics and spatial localisation are therefore useful when anaerobic metabolism is part of the proposed mechanism.

Fungi deserve a place in this discussion. They are easy to miss because routine clinical workflows and many sequencing pipelines focus on bacteria. Yet chronic wound studies have reported fungal communities that are prevalent, dynamic and associated with delayed healing (Kalan et al., 2016). Reviews of the wound microbiome also note that bacteria, fungi and viruses can all influence repair, even though fungal evidence remains less developed than bacterial evidence (White et al., 2023; Uberoi et al., 2024). Fungi may participate in mixed biofilm architecture, alter local metabolism or occupy ecological space after repeated antibacterial exposure; fungal biofilms have been highlighted as an under-recognised part of chronic wound biology (Short et al., 2023). For DFUs, the role of fungi is still not well mapped, but excluding them risks simplifying the niche too much.

The microbial layer therefore has at least four dimensions: taxonomic identity, strain or functional potential, viable activity and spatial organisation. A study that captures only one dimension may still be useful, but it should not be overinterpreted. A genus-level 16S signal may suggest a hypothesis; it does not by itself prove tissue invasion, biofilm activity or host-cell interaction. Conversely, a culture isolate may be clinically important but may not describe the broader community structure surrounding it. Mechanistic DFU microbiology requires these layers to be read together.

## 5 Host repair programs locked by microbial niches

Microbial products do not act on an empty background. They are received by host cells that may already be hypoxic, senescent, metabolically stressed or surrounded by degraded matrix. The same bacterial product that triggers a short protective response in an acute wound may sustain damaging inflammation in a diabetic wound. Host cell state is therefore the missing link between microbial detection and clinical non-healing.

### 5.1 Keratinocytes: re-epithelialisation fails as a cell-state problem

Keratinocytes drive re-epithelialisation. In normal healing, they shift from barrier maintenance to migration and advance from the wound edge, a process requiring coordinated adhesion, cytoskeletal remodelling, matrix interaction and growth-factor signalling (Pastar et al., 2014). In DFUs, this shift can be interrupted by hyperglycaemia, reactive oxygen species, protease excess, inflammatory cytokines and microbial products. Keratinocytes may express chemokines and antimicrobial genes but still fail to move efficiently. Failed re-epithelialisation is therefore not just slow growth. It is an edge-state problem in which microbial signals, degraded matrix and cellular stress disrupt the behaviour needed for closure.

The epithelial edge is a particularly important spatial compartment. A microbial signal in necrotic tissue or wound fluid is not equivalent to a signal positioned next to stalled keratinocytes. Spatial methods should therefore ask whether microbial molecules, biofilm structures, neutrophil products and degraded matrix accumulate near the leading edge. If they do, epithelial arrest may be a direct output of the niche rather than a generic feature of diabetes.

### 5.2 Fibroblasts: matrix reconstruction stalls in inflammatory niches

Fibroblasts sit at the centre of matrix repair. They organise provisional matrix, regulate collagen deposition and communicate with immune, endothelial and epithelial cells. Single-cell analysis of DFU tissue shows that fibroblast states are heterogeneous and clinically relevant (Theocharidis et al., 2022). Healing-associated fibroblast programs involve ECM remodelling, inflammatory signalling and hypoxia responses. More recent single-cell analysis of debrided DFU tissue suggests that patient context and local wound environment can shape fibroblast repair programs (Choi et al., 2025). Fibroblast failure, then, is not simply reduced collagen production. It is a shift toward inflammatory, stress-associated or senescent states that cannot rebuild a stable matrix.

This matters for host-microbe interaction because matrix degradation is both a consequence and a driver of microbial persistence. An inflammatory fibroblast state can sustain chemokines and proteases; proteases damage matrix scaffolds; damaged matrix exposes microbial adhesion sites and releases DAMPs. The loop is likely to differ across wound compartments. Fibroblasts in the wound edge, granulation tissue and deep debrided tissue may not have the same state or the same microbial neighbours.

### 5.3 Myeloid cells: clearance persists but resolution fails

Myeloid cells decide whether inflammation clears or lingers. Neutrophils are needed for early microbial control, but excessive NET formation can injure tissue through histones, elastase and oxidants. Disulfiram was reported to accelerate DFU healing by blocking NET formation through suppression of the NLRP3/caspase-1/GSDMD pathway (Yang et al., 2023). This finding fits a broader interpretation of DFUs as immunologically inefficient rather than immunologically silent. Neutrophil activity continues, tissue damage accumulates and microbial clearance remains incomplete.

Macrophages are expected to clear debris and then support repair (Krzyszczyk et al., 2018). In diabetic wounds, they may instead show impaired phagocytosis, altered metabolic programming and poor transition toward repair-supportive states (Clayton et al., 2024). Persistent stimulation by biofilm, necrotic tissue, hyperglycaemia and DAMPs can keep NF-κB and inflammasome signalling active. Cytokine release continues, while angiogenesis, matrix deposition and epithelial closure are delayed. Keratinocyte arrest, inflammatory fibroblast remodelling, NET excess and macrophage resolution failure should therefore not be read as separate abnormalities. They are different outputs of the same local niche.

### 5.4 Endothelial and vascular repair: hypoxia is a spatial amplifier

Vascular dysfunction is usually discussed as a systemic or limb-level problem, but it also shapes microniches within the wound. Reduced perfusion decreases oxygen delivery, immune-cell trafficking and antibiotic penetration. Endothelial cells exposed to diabetic stress may show impaired migration, proliferation and angiogenic signalling. The result is a patchwork of oxygen and nutrient gradients rather than a uniform wound bed. Microbial communities can exploit those gradients, and host cells located in hypoxic compartments may be less able to mount effective clearance or repair responses.

This is one reason why purely microbial biomarkers are unlikely to be robust across DFUs. A microbial community associated with non-healing in a poorly perfused wound may not have the same significance in a well-perfused neuropathic ulcer. Mechanistic studies should therefore align microbial sampling with perfusion data, wound depth and histological compartments.

### 5.5 Cross-compartment coupling: why location changes meaning

The same microbial profile can have different biological meanings depending on where it sits. A polymicrobial signal in superficial exudate may reflect transient contamination, whereas the same signal embedded in necrotic ECM or positioned near a stalled epithelial edge may indicate a repair-relevant niche. Host cells also differ by compartment. Keratinocytes at the edge, fibroblasts in granulation tissue, myeloid cells in necrotic zones and endothelial cells around poorly perfused regions do not receive the same microbial or metabolic signals. This is why DFU studies should avoid treating the wound as a single homogeneous sample when the question is mechanistic interaction.

Cross-compartment coupling also explains why debridement can produce variable biological effects. Removing necrotic tissue can reduce microbial shelter and DAMP load, but it may also transiently change oxygen exposure, bleeding, tissue architecture and community composition. A study that samples only before and after debridement without mapping compartments may conclude that the microbiome is unstable, while the more important result may be that the location of microbial activity has changed. In a niche-based framework, treatment response should be interpreted as a spatial reorganisation of host and microbial signals, not only as a fall in total bacterial burden.

For clinical translation, this means that a wound may require different priorities at different depths or time points. The surface may need exudate control and biofilm disruption; the edge may need protection of epithelial migration; the bed may need matrix stabilisation; a deeper ischaemic compartment may need vascular optimisation. This layered interpretation is more demanding than a single culture result, but it better matches the biology of non-healing DFUs.

## 6 PAMP-DAMP amplification and the chronic inflammatory loop

The host-microbe interface in DFUs is often discussed through pathogen-associated molecular patterns: lipopolysaccharide, lipoteichoic acid, peptidoglycan and microbial nucleic acids. These signals activate Toll-like receptors, NOD-like receptors, inflammasomes and downstream pathways such as NF-κB, MAPK, IL-1β and IL-18 (Mu et al., 2022; Teh et al., 2023). But PAMPs alone do not explain why inflammation can persist after debridement or antimicrobial therapy. Damaged host tissue supplies its own danger signals, including mitochondrial DNA, extracellular nucleic acids, oxidised lipids and ECM fragments. These DAMPs can keep innate immune pathways active even when microbial load has fallen.

The combination matters. Microbes provide exogenous danger signals; necrotic cells, damaged matrix and senescent cells provide endogenous ones. When both sets of signals remain, the wound has no clear biological message that the danger phase has ended. Inflammation stays open, proteases continue to degrade matrix and repair cells receive mixed instructions. This helps explain why antibiotics alone may not close a chronic DFU. Unless necrotic tissue, mechanical stress, poor perfusion and matrix destruction are addressed, the host side of the loop may continue.

This framework also shows why the clinical distinction between colonisation and infection is not always enough for mechanistic research. A wound may not meet criteria for severe infection but may still contain microbial products and damaged host signals that maintain local inflammation. Conversely, a clinically infected wound may heal if source control and vascular optimisation disrupt the niche. Studies of host-microbe interaction should therefore separate overt infection from biological coupling. The latter requires evidence of microbial activity, tissue location and host response, not just a positive culture or sequencing read.

A useful way to read the loop is to ask what barrier is self-renewing. In some wounds the dominant renewing barrier may be microbial shelter in necrotic tissue or biofilm-like aggregates. In others it may be hypoxia, mechanical re-injury, protease excess or senescence. The same wound can also move between states over time. Longitudinal designs are therefore more informative than one-time comparisons because they can show whether treatment changes only the microbial community, only the host state, or the coupling between them. The capabilities and limitations of the major evidence types are summarized in **Table 2**.

**Table 2:** What different evidence types can and cannot show in DFU host-microbe studies.

Evidence type	What it can support	What it cannot prove alone
Culture and quantitative culture	Clinically actionable organisms and approximate microbial burden.	Non-culturable organisms, biofilm architecture and tissue location.
16S/ITS sequencing and metagenomics	Community composition, fungal or bacterial diversity, strain-level clues and resistance-gene potential.	Microbial viability, transcriptional activity or physical contact with host programs.
Metatranscriptomics/total RNA	A closer view of microbial activity alongside host responses in the same specimen.	Causality; low-biomass signals still need contamination control and validation.
Single-cell transcriptomics	Host cell-state heterogeneity, including keratinocyte stress, inflammatory fibroblasts and myeloid dysfunction.	Native tissue position after dissociation.
Spatial transcriptomics and spatial microbiome approaches	Whether microbial signals, host programs and histological regions co-localise.	Direct mechanism unless paired with perturbation, time course or orthogonal imaging.
Longitudinal cohorts and intervention studies	Temporal order and treatment-associated niche changes.	General causality when confounding by perfusion, antibiotics and offloading is not handled.

## 7 Multi-omics evidence and its boundaries

High-throughput methods have changed DFU microbiology, but no single platform answers the whole question. Culture identifies organisms that matter clinically, yet misses non-culturable communities and biofilm architecture. Quantitative culture or molecular load gives a useful burden estimate, and microbial load above certain thresholds has been associated with poorer outcomes (Soldevila-Boixader et al., 2022). Still, burden does not show whether microbes are organised in tissue or transcriptionally active.

16S rRNA sequencing, ITS sequencing and metagenomics describe community composition, potential functions and sometimes strain-level variation. They are valuable for cohort-level pattern discovery. Their limitation is equally clear: they cannot reliably distinguish live from dead organisms, active from inactive microbes or tissue-adherent organisms from surface or reagent contaminants. Metatranscriptomics and total RNA sequencing move closer to activity because they capture expressed microbial and host transcripts. In diabetic foot infections, a multi-omics strategy linked microbial activity with host inflammatory and stress responses (Radzieta et al., 2021). Even so, low microbial biomass, high host RNA background and contamination remain unavoidable concerns.

Several practical biases deserve particular attention. Amplicon and metagenomic count matrices are compositional: an apparent increase in one taxon can reflect a true increase, a decrease in another taxon or a change in total microbial load. Rarefaction and simple library-size normalisation can therefore distort differential abundance tests when microbial biomass varies across wounds (McMurdie and Holmes, 2014; Gloor et al., 2017; Vandeputte et al., 2017). Host-dominated total RNA and metatranscriptomic datasets add another problem: microbial reads may be rare, so normalising only within the microbial fraction may exaggerate weak signals, whereas normalising against total host-plus-microbial RNA may mask low-abundance but active organisms. Batch effects from extraction kits, reagent lots, sequencing runs, debridement timing, transport and storage can also create apparent wound signatures, especially in low-biomass specimens (Salter et al., 2014; Eisenhofer et al., 2019). Commercial spatial platforms add a sensitivity floor: absence of a microbial transcript in a spatial spot should not be treated as true biological absence unless capture efficiency, probe design, negative controls and orthogonal validation are considered. These issues directly affect whether a taxon is interpreted as a niche driver or as a normalisation artefact.

Single-cell transcriptomics adds host-cell resolution. It can identify keratinocyte, fibroblast, immune and endothelial states that bulk RNA-seq blends together. In DFUs, single-cell data have already revealed altered fibroblast and immune programs in non-healing tissue (Theocharidis et al., 2022). The cost is loss of tissue position. Spatial approaches repair part of that loss by placing host states, microbial signals and histological regions in a shared coordinate system. Sounart et al. (2023) is retained here because it provides a useful precedent: dual spatially resolved transcriptomics was used to examine host-pathogen colocalisation in FFPE tissue. Spatial host-microbiome sequencing and spatial metatranscriptomics extend the same logic by linking microbial communities with host tissue niches (Lotstedt et al., 2024; Saarenpaa et al., 2024).

Computation is useful only if it follows a biological question. Squidpy can support spatial neighbourhood analysis and spatial autocorrelation (Palla et al., 2022). cell2location, RCTD and CARD can estimate cell type composition in spatial transcriptomic data using single-cell references (Cable et al., 2022; Kleshchevnikov et al., 2022; Ma and Zhou, 2022). CellChat, NicheNet and SpaTalk can infer ligand-receptor communication and spatial interactions (Browaeys et al., 2020; Jin et al., 2021; Shao et al., 2022). For DFUs, the questions should come first: are microbial signals located in necrotic, hypoxic or biofilm-like regions; are host responses distance-dependent; and are NETs, MMPs, inflammatory fibroblasts or senescent programs enriched near microbial hotspots? Without that structure, multi-omics can easily produce attractive figures without mechanistic traction.

A rigorous DFU multi-omics study should therefore report what the sample represents. Swabs, curettage, debrided tissue, wound-edge biopsy and deep tissue biopsy are not interchangeable. The study should also include negative extraction controls, reagent blanks, sample handling details and microbial-load measures whenever possible. Sequencing depth alone cannot rescue an uninterpretable sample. For spatial designs, histological annotation of necrosis, granulation tissue, wound edge, vessel density and biofilm-like material is as important as the sequencing platform itself.

### 7.1 Minimal evidence chain for claiming host-microbe interaction

The evidence chain for host-microbe interaction should be stated explicitly. At the weakest level, a study can show association: a taxon, functional gene or microbial community pattern differs between healing and non-healing wounds. This is useful for hypothesis generation but does not prove that microbes are active or repair-relevant. A stronger level is activity: viable culture, microbial RNA, expressed virulence functions, microbial load or biofilm matrix suggests that the signal is biologically alive. Stronger still is spatial coupling: microbial activity is located near a host program such as epithelial arrest, NET accumulation, MMP excess, hypoxia or senescence. The strongest evidence combines spatial coupling with temporal order or perturbation, showing that the niche changes before or during a change in wound trajectory.

This hierarchy is important because multi-omics can make weak evidence look visually persuasive. A heat map of differentially abundant genera and a volcano plot of host genes may be attractive, but they remain parallel associations unless the data show that the microbial and host signals interact in the same tissue space or time window. Spatial neighbourhood analysis, distance-to-feature tests and matched histology can help, but even these should be treated cautiously when sampling is sparse or tissue architecture is disrupted.

A publishable high-impact DFU omics study therefore needs methodological modesty. It should distinguish discovery from validation, report limitations of low-biomass microbial data, and avoid claiming causality from taxonomy alone. For the present review, the same principle shapes the interpretation: microbial niches are proposed as a testable framework, not as a settled universal mechanism for every DFU.

### 7.2 Testable predictions and falsifiability of the pathological niche model

The niche model makes several discriminating predictions. First, microbial activity should not be randomly distributed across the wound; it should be enriched in tissue compartments compatible with persistence, such as necrotic matrix, exudative pockets, biofilm-like aggregates, poorly perfused zones or interfaces near a stalled epithelial edge. Second, the neighbouring host response should be non-random: NET markers, inflammasome signalling, MMP excess, SASP-related chemokines, inflammatory fibroblast states, epithelial arrest or endothelial dysfunction should be more pronounced near microbial activity than in histologically comparable regions with little microbial signal. Third, if the coupling is mechanistically relevant, some responses should show distance dependence; the strength of host inflammatory or repair-arrest programs should change with proximity to microbial hotspots after accounting for wound compartment, perfusion and necrosis. Fourth, a treatment that disrupts the niche should reduce microbial activity, DAMP-rich tissue or biofilm-like structure before or alongside recovery of host repair programs and an improved wound trajectory.

The framework is also falsifiable. It would be weakened if microbial signals were only superficial or randomly distributed, if host non-healing programs remained unrelated to microbial activity after controlling for perfusion, necrosis, pressure and antibiotic exposure, or if microbial activity changed substantially without any corresponding spatial, temporal or clinical change in host repair state. A particularly important alternative hypothesis is that perfusion failure alone generates both microbial persistence and repair failure as parallel downstream consequences. This alternative should be named rather than hidden as confounding. Studies can discriminate between the two models by comparing compartments with similar hypoxia but different microbial activity, by testing whether host programs vary with distance from microbial hotspots within the same perfusion stratum, or by following whether debridement, antimicrobial intervention or revascularisation changes the microbial-host coupling rather than only one arm of the response.

## 8 Translational implications: from organisms to niche-based stratification

The niche framework has practical implications. Debridement, for example, is not only mechanical cleaning. It removes microbial shelter, DAMP-rich material and unstable tissue surfaces. Offloading limits repeated injury that can rebuild the niche. Vascular assessment and revascularisation, when indicated, improve oxygen delivery and immune competence. These interventions are microbiologically relevant even though they are not antibiotics.

The same logic argues against single-target treatment thinking. Antimicrobial therapy may reduce PAMP input, but DAMPs, senescence, protease excess and poor perfusion can keep inflammation active. Regenerative products may also fail if microbial burden, necrotic tissue and hypoxia remain uncontrolled. A more useful approach is to ask what dominates in a particular wound: microbial persistence, perfusion failure, mechanical injury, necrotic tissue, excessive protease activity or repair arrest. The treatment combination should follow that dominant barrier rather than a generic wound label.

Biomarkers should probably be read as profiles. A single genus, cytokine or gene is unlikely to predict DFU outcome across heterogeneous patients. More informative profiles may combine microbial burden, biofilm likelihood, infection severity, perfusion status, wound duration, inflammatory state and repair programs. Longitudinal sampling is especially important. A one-time sample may miss the transition from a contaminated but repairable wound to a stable non-healing niche.

The framework also leaves room for ecological therapy, although the clinical evidence is still early. The goal may not always be total microbial eradication. In some settings, it may be more realistic to disrupt biofilm shelter, reduce pathogenic activity, restore host clearance and permit repair-compatible communities. The observation that some wound-associated bacteria can promote re-epithelialisation under specific conditions supports this possibility (White et al., 2024). For DFU care, this remains a research direction rather than a ready clinical prescription.

A niche-based profile should never replace standard clinical assessment. Infection severity, vascular status, offloading, glycaemic management, surgical source control and patient safety remain primary. The added value of the niche framework is that it can explain why a technically correct intervention may fail if it addresses only one side of the loop. A wound with bacterial activity in necrotic tissue, high DAMP burden and poor perfusion may require staged source control and vascular optimisation before repair-directed treatment is likely to work. A wound with modest microbial burden but high protease activity and senescent fibroblast signals may need a different research strategy.

### 8.1 From stratification to treatment sequencing

The most useful clinical implication of the niche model is treatment sequencing. In a wound with severe infection, abscess, necrosis or systemic risk, source control and guideline-directed antimicrobial therapy remain the priority. In a wound with inadequate perfusion, however, repeated antimicrobial courses or advanced dressings may have limited durability until oxygen delivery and tissue viability are improved. In a wound with repeated callus and pressure injury, the ecological problem is rebuilt by trauma unless offloading is effective. Thus, the niche framework does not add another isolated therapy; it helps decide which barrier must be removed first.

Repair-directed treatments should be interpreted in the same way. Growth factors, matrices, skin substitutes, negative-pressure therapy or other adjunctive approaches may fail if placed into a wound bed dominated by microbial shelter, necrotic DAMPs, high protease activity or unresolved hypoxia. Conversely, a wound that has undergone adequate source control and vascular optimisation may still fail because fibroblast, epithelial or myeloid programs remain locked in a chronic inflammatory state. In that setting, research biomarkers could help identify when a repair-directed intervention is biologically plausible.

Niche-based stratification may also improve trial design. Many DFU trials enrol heterogeneous wounds and then test one intervention against a mixed set of barriers. A therapy that helps a protease-dominant wound may appear ineffective if the cohort contains many ischaemia-dominant or biofilm-dominant wounds. Future trials could use simple clinical strata first, then add microbial load, inflammatory state, matrix/protease markers or spatial features as enrichment tools. Such designs would not require every clinic to perform spatial omics; rather, omics studies would define the biology behind practical trial subgroups.

**Table 3** should be read as a conceptual stratification scheme rather than a validated clinical classifier. The five patterns were derived from the pathological barriers and evidence types reviewed above - microbial persistence and biofilm tolerance, perfusion or hypoxia, protease-driven ECM degradation, cellular senescence and immune-resolution failure - not from a new reanalysis of existing DFU cohorts. They are intended to help formulate hypotheses, enrich future trials and interpret mixed omics data.

**Table 3:** Proposed niche-based DFU patterns for research stratification and therapeutic reasoning.

Pattern	Likely dominant barrier	Suggested readouts	Therapeutic logic	Key caution
Biofilm-/persistence-dominant	Biofilm-like aggregates, high viable microbial load, recurrent infection or poor antimicrobial response.	Culture plus load, histology/FISH, metagenomics/metatranscriptomics, debridement response.	Source control, debridement, antimicrobial optimisation and biofilm-disrupting strategies.	Avoid assuming all sequencing reads are active infection.
Ischaemia-/hypoxia-dominant	Poor perfusion limits immune function, oxygen delivery and antibiotic access.	Perfusion testing, vascular imaging, hypoxia markers, spatial localisation of anaerobic or low-oxygen zones.	Vascular optimisation before expecting sustained antimicrobial or regenerative response.	Microbiome associations may be confounded by tissue oxygenation.
Protease-/ECM-degradation-dominant	MMP excess, degraded matrix and exudate maintain tissue instability.	Wound-fluid proteases, ECM fragments, fibroblast states, histological matrix quality.	Debridement, exudate control, matrix protection and repair-directed therapy after source control.	Protease signals may be host-driven even after microbial reduction.
Senescence-dominant	SASP chemokines and proteases maintain inflammation and impair repair.	CDKN1A, CXCL8, IGFBP2, IL1A, MMP10, SERPINE1 and spatial senescence markers.	Research focus on senescence-modulating or matrix-stabilising strategies.	Current evidence is mechanistic and not yet a routine clinical test.
Immune-resolution-failure dominant	NETs, inflammasomes and macrophage dysfunction persist despite visible inflammation.	NET markers, NLRP3/IL-1 axis, myeloid single-cell states, bacterial activity near immune hotspots.	Combine microbial control with resolution-directed and repair-supportive approaches.	Suppressing inflammation without source control may worsen infection risk.

The categories are not mutually exclusive. In practice, poor perfusion, microbial persistence and MMP excess may coexist in the same wound, and a wound may shift from one dominant barrier to another after debridement, antimicrobial therapy or revascularisation. A niche-based assessment should therefore identify a dominant barrier together with important co-dominant barriers and then ask whether the proposed treatment sequence addresses the barrier most likely to rebuild the niche. For example, a wound with poor perfusion, microbial persistence and protease excess would usually require vascular optimisation and source control before a matrix-directed or regenerative therapy could be expected to work. **Table 3** therefore offers a reasoning tool, not a rigid taxonomy. Operational criteria for identifying a pathological microbial niche are summarized in **Table 4**.

**Table 4:** Operational criteria for identifying a pathological microbial niche in DFU studies.

Criterion	Minimum evidence	Why it matters
Location	Tissue biopsy, mapped compartment, histology, FISH, spatial transcriptomics or clearly defined sampling site.	Distinguishes tissue-relevant signal from surface contamination or mixed wound-fluid signal.
Microbial activity	Quantitative burden, culture viability, RNA signal, expressed virulence/resistance features, biofilm matrix or microbial products.	Separates active persistence from DNA remnants or low-level detection.
Host repair state	Neighbouring host programs such as NETs, inflammasomes, MMP excess, fibroblast stress, epithelial arrest, hypoxia or senescence.	Links microbial signal to a plausible non-healing mechanism.
Clinical context	Perfusion, offloading, infection grade, wound duration, antibiotics, debridement, depth and necrosis recorded longitudinally.	Reduces confounding and supports stratified interpretation.
Validation	Orthogonal imaging, spatial statistics, time course, intervention response or experimental perturbation.	Moves the finding from association toward mechanism.

## 9 Unresolved questions and future directions

Causality remains the deepest gap. Much of the current DFU microbiome literature can show that microbial burden, community structure or strain-level variation tracks with outcome. It cannot always tell whether microbes are initiating non-healing, taking advantage of a wound that has already failed to repair, or doing both at once. The next useful step is not another cross-sectional survey by default. Longitudinal sampling, defined microbial communities, targeted depletion or transplantation, and host-pathway perturbation will be needed to separate driver, passenger and amplifier roles. A design checklist for future DFU host-microbe niche studies is provided in **Table 5**.

**Table 5:** Design checklist for future DFU host-microbe niche studies.

Checklist item	Recommended practice
Clinical metadata	Record infection grade, perfusion, wound duration, depth, offloading, antibiotics, debridement and glycaemic context.
Sampling map	Specify wound edge, bed, necrosis, deep tissue, exudate or swab; avoid mixing compartments without annotation.
Microbial signal	Report culture results where clinically relevant, microbial load, sequencing controls and evidence of viability or RNA activity.
Host signal	Profile keratinocytes, fibroblasts, endothelial cells, neutrophils/macrophages, proteases, NETs, inflammasomes and senescence markers.
Spatial validation	Use histology, FISH, targeted *in situ* assays or spatial transcriptomics to test co-localisation.
Longitudinal logic	Repeat sampling around debridement, antimicrobial treatment, vascular intervention or healing/non-healing transition.
Mechanistic restraint	State clearly whether the evidence supports association, spatial coupling, temporal order or causal perturbation.

Spatial evidence is still limited. Swabs and homogenised biopsies flatten wound edge, wound bed, necrosis, granulation tissue and deeper compartments into one signal. That averaging is convenient, but it fits the biology poorly. A microbial signal is more meaningful when it can be placed next to a damaged matrix, a biofilm-like structure, an inflammatory myeloid niche, a stalled epithelial edge or a poorly perfused region. Spatial designs should ask where microbes are, whether they are active there, and what host program is present in the neighbouring tissue.

Microbial activity and contamination control also need more careful handling. DFU samples can be low in microbial biomass relative to host RNA, and they are exposed to dressings, topical agents, antibiotics and the environment. A taxonomic assignment should not be treated as interaction by itself. Stronger studies will combine negative controls, microbial-load information, transcriptional evidence, tissue localisation and, when feasible, orthogonal validation such as staining, FISH or targeted *in situ* assays.

Clinical stratification is better framed as a design problem than as an afterthought. A short-duration neuropathic ulcer, an ischaemic ulcer after repeated antibiotics and a deep infected wound with necrosis may all be called DFU, but their microbial niches are unlikely to be the same. Future studies should avoid treating all wounds as one population when the relevant mechanisms may differ. Biofilm-dominant, ischaemia-dominant, protease-dominant, senescence-dominant and immune-resolution-failure patterns may eventually require different combinations of debridement, antimicrobial treatment, vascular intervention and repair-directed therapy.

A practical study design would recruit patients across clinically defined strata, record treatment exposures and perfusion status, collect spatially matched wound-edge and wound-bed samples, quantify microbial load and activity, profile host-cell states and validate key findings by imaging. The most convincing endpoint would not be a differentially abundant genus alone, but a treatment-responsive niche signature: microbial activity decreases, DAMP-rich tissue is removed, host repair programs recover and the wound trajectory changes.

## 10 Conclusion

DFU non-healing is better understood as a local ecological failure than as a simple accumulation of hyperglycaemia, ischaemia, infection and inflammation. These factors create wound regions in which microbial persistence and host repair failure reinforce each other. Hyperglycaemia, hypoxia, necrotic tissue, ECM breakdown and senescence provide ecological and structural support for microbial communities. Microbial products, biofilm organisation and endogenous danger signals then sustain NET formation, inflammasome activity, protease release, inflammatory fibroblast states, defective macrophage resolution, impaired angiogenesis and failed re-epithelialisation.

Modern microbiome and multi-omics studies are valuable only if they test this mechanism rather than add more lists of differentially abundant taxa or genes. The key question is whether microbes are active, spatially organised and linked to host non-healing programs. Answering it will require microbial ecology, histopathology, host cell-state analysis, spatial statistics, longitudinal clinical data and experimental validation to be interpreted together. If that evidence chain can be built, DFU research may move from descriptive microbiome association toward actionable niche-based stratification.

## Data Availability

The original contributions presented in the study are included in the article/supplementary material. Further inquiries can be directed to the corresponding author.
